# Predicting Physical Time Series Using Dynamic Ridge Polynomial Neural Networks

**DOI:** 10.1371/journal.pone.0105766

**Published:** 2014-08-26

**Authors:** Dhiya Al-Jumeily, Rozaida Ghazali, Abir Hussain

**Affiliations:** 1 Applied Computing Research Group, Liverpool John Moores University, Liverpool, Mersyside, United Kingdom; 2 Faculty of Computer Science and Information Technology, Universiti Tun Hussein Onn Malaysia, Parit Raja, Batu Pahat, Malaysia; Universiteit Gent, Belgium

## Abstract

Forecasting naturally occurring phenomena is a common problem in many domains of science, and this has been addressed and investigated by many scientists. The importance of time series prediction stems from the fact that it has wide range of applications, including control systems, engineering processes, environmental systems and economics. From the knowledge of some aspects of the previous behaviour of the system, the aim of the prediction process is to determine or predict its future behaviour. In this paper, we consider a novel application of a higher order polynomial neural network architecture called Dynamic Ridge Polynomial Neural Network that combines the properties of higher order and recurrent neural networks for the prediction of physical time series. In this study, four types of signals have been used, which are; The Lorenz attractor, mean value of the AE index, sunspot number, and heat wave temperature. The simulation results showed good improvements in terms of the signal to noise ratio in comparison to a number of higher order and feedforward neural networks in comparison to the benchmarked techniques.

## Introduction

Time series generally refers to a sequence of data points spaced at time intervals and measured typically at successive times. Practically, it is a collection of historical data of one system, such as a stock price, traffic data, and the pollution rates. A time series can be used in two ways for different purposes:

- Looking backwards – the use of historical data to analyze the previous behaviour of a system. Applications include diagnosis or recognition of machine fault or human disease.
- Looking forwards – the use of data to predict or forecast the future behaviour of a system. Applications include stock or price prediction, market demand forecast, and natural data prediction.

Time series analysis comprises of methods that attempt to understand the behaviour of such time series, often either to understand the underlying theory of the data points, or to make forecasts. Time series forecasting is the use of a model to predict future events or future data points based on known past events. It is a process that produces a set of outputs by a given set of historical variables. Forecasting assumes that future occurrences are based on past or present events, in which some aspects of the past patterns will continue into the future. Past relationship can then be discovered through study and observation. In other words, time series forecasting is discovering the relationship between present, past and future observations. According to Plummer [42], the aim of time series forecasting is to observe or model the existing data series which can be in different forms for example financial data series (stocks, indices, exchange rates, etc), physically observed data series (sunspots, weather, etc), and mathematical data series (Fibonacci sequence, integrals of differential equations, etc).

Time series forecasting takes an existing series of data *X_t-n_*, ….., *X_t-2_*, *X_t-1_*, *X_t_* and forecasts *X_t+1_*, *X_t+2_*,….. data values. Theoretically, these series can be seen as a continuous function of time variable *t*. For practical purposes, however, time is usually viewed in terms of discrete time steps. The size of the time interval depends on the problem at hand, and can be anything from milliseconds to hours, days, or even years. If the time series contains only one component, it is called a univariate time series; otherwise it is a multivariate time series. In a univariate series, the input variables are restricted to the signal being predicted, while in multivariate series, the raw data comes from a variety of indicators which will form the actual inputs variable. In a multivariate series, any indicator whether or not it is directly related to the output can be incorporated as the input variable [43].

Traditional methods for time series forecasting are statistics-based, including moving average (MA), autoregressive (AR), autoregressive moving average (ARMA) models, linear regression and exponential smoothing [1]. These methods do not produce fully satisfactory results, due to the nonlinear behaviour of most of the natural occurring time series. Other more advanced techniques such as neural networks [2], [3], fuzzy logic [4] and fractals [5] have been successfully used in time series prediction.

Neural networks (NNs) provide a general class of nonlinear models which have been successfully applied in many engineering and scientific problems. These include real world problems such as Time Series Prediction [35]–[36], speech/character/pattern recognition [37]–[38], system identification, Medical Image Analysis [34], System Optimization, Function Approximation and many more applications. Their numerous application domains fall into many categories: for example regression and generalization, classifications, association, clustering, pattern completion, and optimization.

The idea of artificial neural networks (ANNs) is to model a neuron by building interconnected networks, and devise learning algorithms to work out the ANNs. Often the term ‘Neural networks’ is used as a broad sense which group together different families of algorithms and methods.

The application of neural networks in time series prediction has shown better performance in comparison to statistical methods because of their nonlinear nature and training capability. In addition, it has been shown that neural networks are universal approximators and have the ability to produce complex nonlinear mappings [6].

Neural networks can be divided into two major types, feedforward and recurrent networks. Feedforward neural networks, such as the multilayer perceptron (MLP) and the radial basis function (RBF) neural network, have been successfully used for time series prediction [7]. However, MLPs utilise computationally intensive training algorithms (such as error back-propagation [8]) and can get stuck in local minima. In addition, these networks have problems in dealing with large amounts of training data, while demonstrating poor interpolation properties, when using reduced training sets. In the case of RBFs, the networks are trained only once on a large example set taken from the signal such that the dynamics of the underlying system can be captured. Therefore, the networks produce sequential outputs in response to newly arriving data. Therefore, the system can be used when the dynamics of the time series does not change considerably over time, a condition which is usually contravened in practice [7]. Recurrent neural networks have advantages over feedforward networks in that they have the ability to store previous state information and prevent the need to predict the model order of the time series [9].

Despite the encouraging results of using artificial neural networks for time series prediction compared to linear statistical models, the robustness of these findings has been questioned [10], due to a number of well-known problems with neural models such as:

1. Using the same data set, various neural network architectures can produce different results. The main reason for this inconsistency in the results relates to the fact that there are different classes of decision boundaries which different ANN's prefer. Multilayer perceptrons, radial basis functions networks and self-organizing maps when they are trained and tested for the same database can produce different results since they have different topologies [11].
2. Neural network architectures suffer from overfitting and as a result, the size of the network, learning parameters and training data have to be selected experimentally and carefully in order to achieve good generalisation, which is critical when using the network for temporal time series prediction.
3. The inherent nonlinearity and nonstationary of natural time series can prevent a single neural network from being able to accurately forecast changes in the training and the testing data.

To overcome the problems associated with neural networks when used for time series forecasting; in this paper, a novel application of the Dynamic Ridge Polynomial Neural Networks (DRPNN) [39] is proposed for the prediction of physical time series in which the size of the network will be changed during the learning process using a constructive learning method. The network will start with a small basic structure, which will grow as the learning process proceeds until the required approximation error is achieved.

Feedforward neural networks are Nonlinear Autoregressive (NAR) models, on the other hand recurrent neural networks are nonlinear autoregressive moving average models (NARMA). This means that recurrent neural network have advantages over feedforward neural network, similar to the advantages in which autoregressive moving average (ARMA) model posses over Autoregressive (AR) model [15].

## Methods

### 1. Higher Order Neural Networks (HONNs)

Although most neural network models share a common goal in performing functional mapping, different network architectures may vary significantly in their ability to handle different types of problems. For some tasks, higher order combinations of some of the inputs or activations may be appropriate to help form good representations for solving problems. Higher Order Neural Networks (HONNs) are needed because ordinary feedforward network like Multilayer Perceptrons (MLPs) cannot elude the problem of slow learning, especially when involving highly complex nonlinear problems [16].

HONNs distinguish themselves from ordinary feedforward networks by the presence of high order terms in the network. In a great number of Neural Networks models, neural inputs are combined using the summing operation. HONNs contain not only summing unit, but also units that multiply their inputs which are referred to as high order terms or product units. These high order terms or product units can increase the information capacity of a network compared to the networks that have summation units only. The larger capacity means that the same function or problem can be solved by network that has fewer units. HONNs also make use of non-linear interactions between the inputs. The networks therefore expand the input space into another space where linear separability is possible [17].

This section is concerned with introducing a few types of HONNs; Functional Link Neural Network, Pi-Sigma Neural Network, and Ridge Polynomial Neural Network. Each one of them employs the powerful capabilities of product units with some combinations with summing units. Their architectures vary the position where the product units or higher-order terms are used in the networks. The Functional Link Neural Network utilizes the higher-order terms at the input layer as inputs to the network in addition to the original raw inputs. For the Pi-Sigma Neural Network, the existence of the product unit in the network is at the output layer, as the output of the network itself. The third HONN model, the Ridge Polynomial Neural Network made the higher order terms available as the whole hidden layer of product units feeding into a subsequent layer of summing units. All these HONNs models have only one layer of tuneable weights, resulting in simple weights updating procedure in their training.

#### 1.1 Functional Link Neural Network (FLNN)

FLNN was first introduced by Giles and Maxwell [18]. It naturally extends the family of theoretical feedforward network structure by introducing nonlinearities in inputs patterns enhancements [19]. These enhancement nodes act as supplementary inputs to the network. FLNN calculates the product of the network inputs at the input layer, while at the output layer the summations of the weighted inputs are calculated.

FLNN can use higher order correlations of the input components to perform nonlinear mappings using only a single layer of units. Since the architecture is simpler, it is supposed to reduce computational cost in the training stage, whilst maintaining good approximation performance [20]. A single node in the FLNN model could receive information from more than one node by one weighted link. The higher order weights, which connect the high order terms of the input products to the upper nodes have simulated the interaction among several weighted links. For that reason, FLNN could greatly enhance the information capacity and complex data could be learnt [20]–[22].

**Figure 1:**
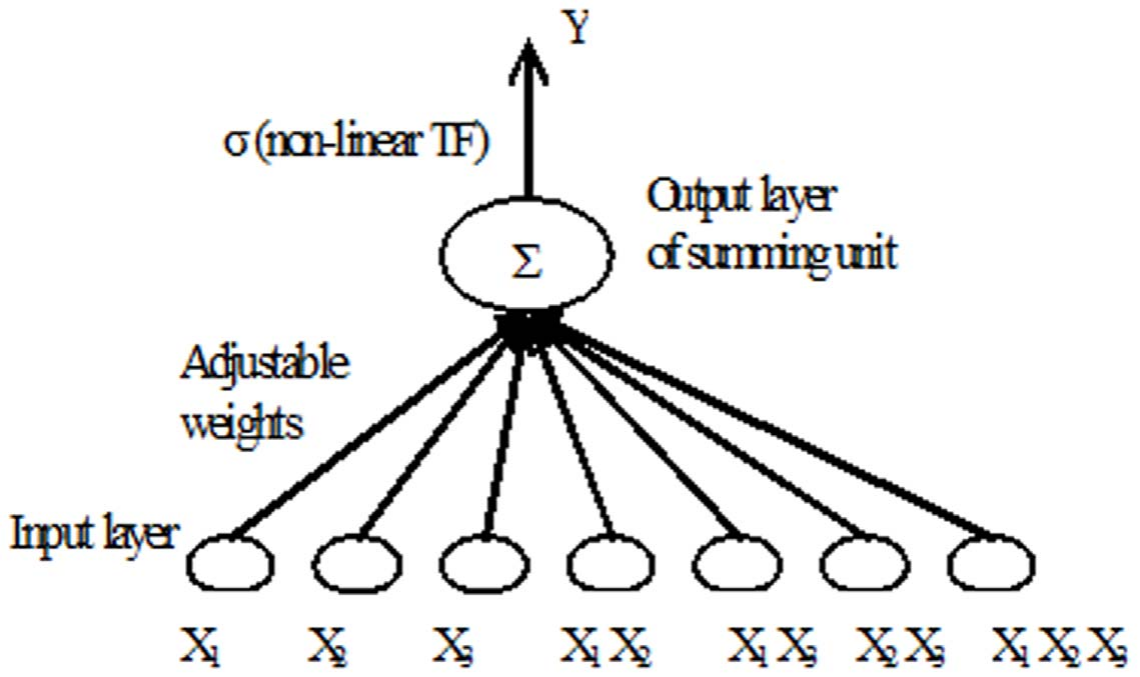
Functional Link Neural Network.

Fei and Yu [23] showed that FLNN has a more powerful approximation capability than conventional Backpropagation networks, and it is a good model for system identification [20]. Cass and Radl [21] used FLNN in process optimization and found that FLNN can be trained much faster than MLP network without scarifying computational capability. FLNN has the properties of invariant under geometric transformations [19]. The model has the advantage of inherent invariance, and only learns the desired signal. Figure [1] shows an example of third order FLNN with three external inputs *x_1_, x_2_*, and *x_3_*_,_ and four high order inputs which act as supplementary inputs to the network.

The output of FLNN is determined as follows: 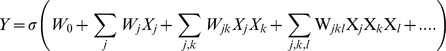
where σ is a nonlinear transfer function, and *w_o_* is the adjustable threshold. Unfortunately, FLNN suffers from the explosion of weights which increase exponentially with the number of inputs. As a result, second or third order functional link networks are considered in practice [24], [25].

#### 1.2 Pi-Sigma Neural Network (PSNN)

PSNN was first introduced by Shin and Ghosh [26]. It is a feedforward network with a single ‘hidden’ layer and product units in the output layer [27]. PSNN calculates the product of the summing units at the output layer and pass it to a nonlinear function. PSNN is able to learn in a stable manner even with fairly large learning rates [28]. The use of linear summing units makes the convergence analysis of the learning rules for the PSNN more accurate and tractable.

**Figure 2:**
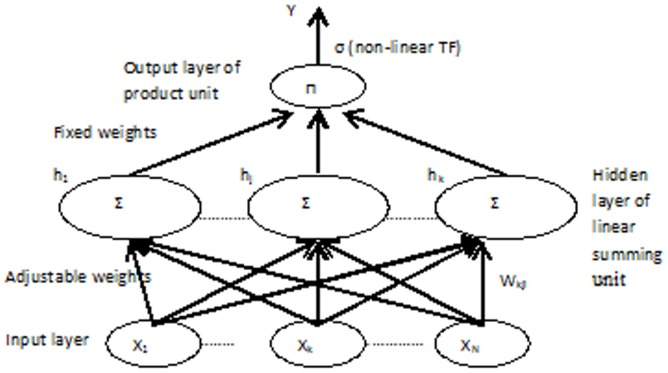
Pi-Sigma Neural Network of *K*-th order.

Previous research found that the Pi-sigma neural network is a good model for various applications. Shin and Ghosh [28] investigated the applicability of PSNN for shift, scale and rotation invariant pattern recognition. Results for both function approximation and classification were extremely encouraging when compared to backpropagation for achieving similar quality solution. Again, Ghosh and Shin [26] argued that PSNN requires less memory (weights and nodes), and at least two orders of magnitude less number of computations when compared to MLP for similar performance level, and over a broad class of problems. Figure [2] shows the Pi-Sigma Neural Network structure with a single output.

The output of the Pi-sigma Network is computed as follows:
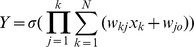
where *w_kj_* is the adjustable weight, *x_k_* is the input vector, *K* is the number of summing unit, *N* is number of input nodes, and 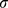 is a suitable nonlinear transfer function. PSNN demonstrated competent ability to solve scientific and engineering problems [26]–[28], however the networks are not universal approximator.

#### 1.3 Ridge Polynomial Neural Network (RPNN)

**Figure 3:**
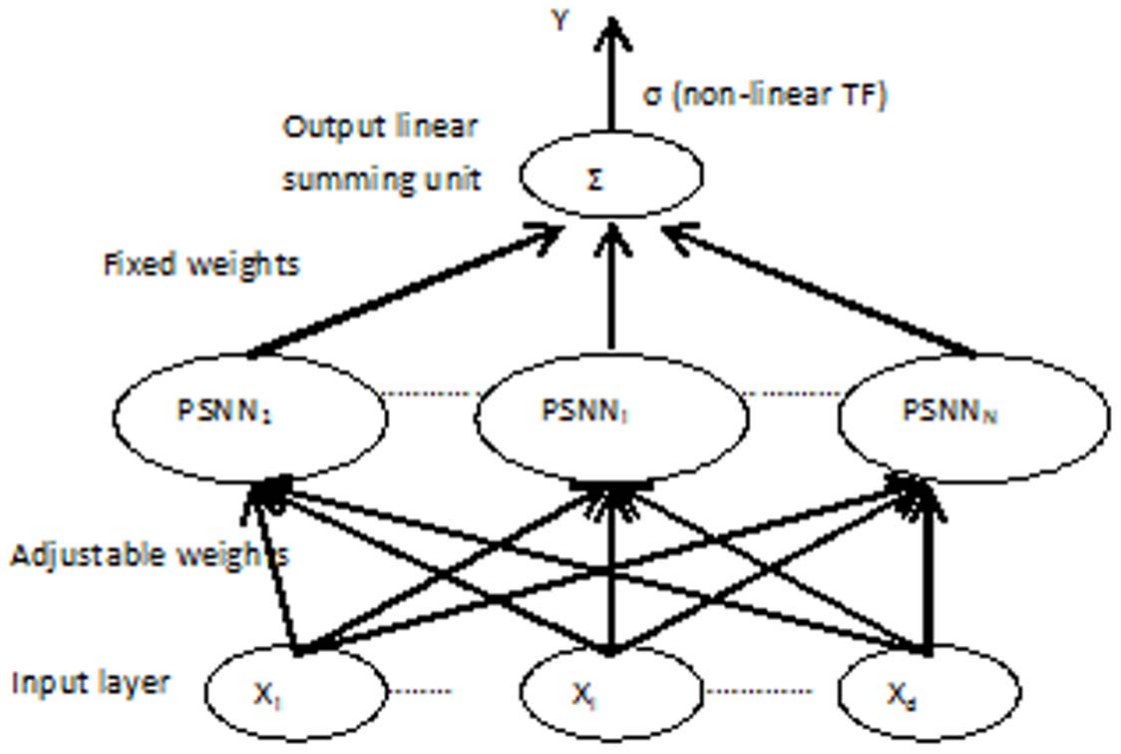
Ridge Polynomial Neural Network of N-th.

RPNNs were first introduced by Shin and Ghosh [12]. They are generalizations of the Pi-Sigma Neural Networks. RPNNs are constructed by adding different degrees of PSNN as a basic building block as shown in Figure [3]. They utilise univariate polynomials and provide efficient and regular structure in comparison to ordinary higher-order feedforward networks [12]. RPNN can approximate any multivariate continuous functions on a compact set in multidimensional input space, with arbitrary degree of accuracy. Similar to the PSNN neural networks, RPNN has only a single layer of adaptive weights and they preserve all the advantages of PSNN.

The output of Ridge Polynomial Neural Network is determined as follows:
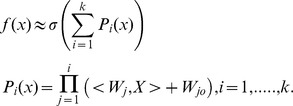
where <*W_j_, X*> is the inner product between the trainable weights matrix *W*, and the input vector *X*. *k* is the number of PSNN blocks used, and 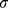 denotes a suitable nonlinear transfer function.

RPNN provides a natural mechanism for incremental network growth, by which the number of free parameters is gradually increased with the addition of Pi-Sigma units of different orders. Unlike other growing networks such as self-organising neural networks (SONN) and group methods of data handling (GMDH) [12], in which their structure have the capability of growing to any arbitrary number of hidden layers and nodes, RPNN has a well regulated architecture. The network can be incrementally grown with the orderly architecture and the network decides which higher order terms are necessary for the task at hand.

Tawfik and Liatsis [29] have tested the RPNN for one step prediction of the Lorenz attractor and solar spot time series. They proved that RPNN has a more regular structure and superior performance in terms of speed and efficiency when compared to Multilayer Perceptron. Voutriaridis, *et.al* [30] found that RPNN could give satisfactory results when used in function approximation and character recognition.

### 2. Dynamic Ridge Polynomial Neural Networks (DRPNNs)

In this section, the structure of the recurrent ridge polynomial neural network will be shown [40]. Feedforward HONNs can only implement a static mapping of the input vectors. In order to model dynamical functions of the brain, it is essential to utilize a system that is capable of storing internal states and can implement complex dynamics. Neural networks with recurrent connections are dynamical systems with temporal state representations. Because of their dynamic structure, they have been successfully used to solve a variety of problems.

#### 2.1 The Properties and Network Structure of DRPNNs

**Figure 4:**
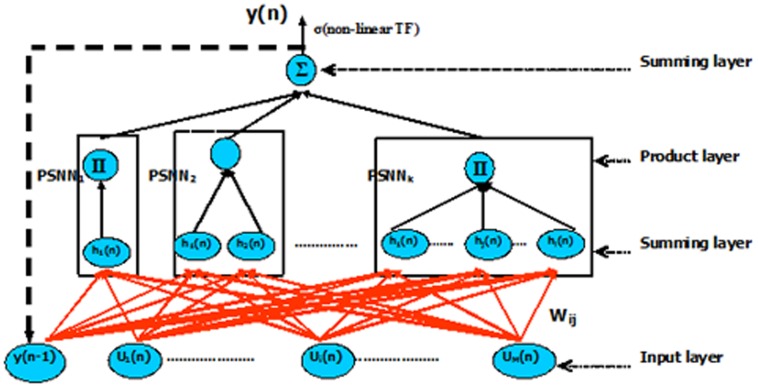
Dynamic Ridge Polynomial Neural Network of *k*-th order.

The structure of the DRPNN is constructed from a number of increasing order Pi-Sigma units with the addition of a feedback connection from the output layer to the input layer. The feedback connection feeds the activation of the output node to the summing nodes in each Pi-Sigma units, thus allowing each building block of Pi-Sigma unit to see the resulting output of the previous patterns. In contrast to RPNN, the DRPNN, as shown in Figure [4], is provided with memories which give the network the ability to retain information to be used later. All the connection weights from the input layer to the first summing layer are learnable, while the rest are fixed to unity.

Consider a DRPNN with *M* number of external inputs *U(n)*, and let *y(n-1)* to be the output of the DRPNN at previous time step. The overall input to the network are the concatenation of *U(n)* and *y(n-1),* and is referred to as *Z(n)* where:
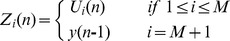
(1)


The output of the *k_th_* order DRPNN is determined as follows:
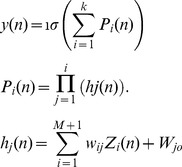
(2)where *k* is the number of Pi-Sigma units used, *P_i_(n)* is the output of each PSNN block, *h_j_(n)* is the net sum of the sigma unit in the corresponding PSNN block, *W_jo_* is the bias, and σ is the sigmoid activation function.

#### 2.2 Learning Algorithm of DRPNN

The DRPNN uses a constructive learning algorithm based on the asynchronous updating rule of the Pi-Sigma unit. The network adds a Pi-Sigma unit of increasing order to its structure when the difference between the current and the previous errors is less than a predefined threshold value. DRPNN follows the following steps for updating its weights [40]:

1. Start with low order DRPNN
2. Carry out the training and update the weights asynchronously after each training pattern.
3. When the observed change in error falls below the predefined threshold r, i.e., 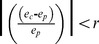 , a higher order PSNN is added. Note that *e_c_*is the Mean Squared Error (MSE) for the current epoch, and*e_p_*is the MSE for the previous epoch.
4. The threshold *r*, for the error gradient and the learning rate*n*, are reduced by a suitable factor respectively.
5. The updated network carries out the learning cycle (repeat steps 1 to 4) until the maximum number of epoch is reached.

The weights of the Pi-Sigma unit in the DRPNN are updated using the Real Time Recurrent Learning algorithm [13]. Instead of modifying all weights synchronously at each update step, in this learning algorithm, we choose only one subset of weights (weights that belong to the latest added PSNN) to tune at a time. A standard error measure used for training the network is the Sum Squared Error: 
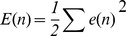
(3)


The error between the target and actual signal is determined as follows: 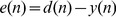
(4)where *d(n)* is the target output at time *n*, *y(n)* is the forecast output at time *n*.

At every time *n*, the weights are updated according to:
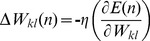
(5)where 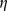 is the learning rate. The value 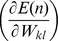
is determined as:



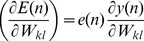
(6)

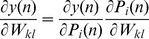
(7)where



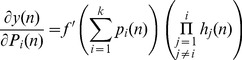
(8)

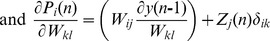
(9)where 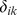 is the Krocnoker delta. Assume *D* as the dynamic system variable (the state of the *ij^th^* neuron), where *D* is:



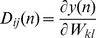
(10)The state of a dynamical system is formally defined as a set of quantities that summarizes all the information about the past behaviour of the system that is needed to uniquely describe its future behaviour [33]. Substituting Equation (8) and (9) into (7) results in:
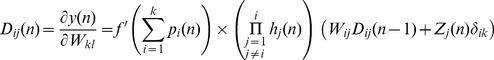
(11)


For simplification, the initial values for *D_ij_(n-1) = 0*, and *Z_j_(n-1) = 0.5.* Then the weights updating rule is
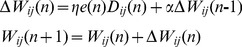
(12)


### 3. Time Series Prediction Using Dynamic Ridge Polynomial Neural Network

#### 3.1 Time series Used in the Experiments

Four time series have been used for our experiments, namely the Lorenz attractor, the Mean value of the AE index, sunspot number, and heat wave temperature time series.

**Figure 5:**
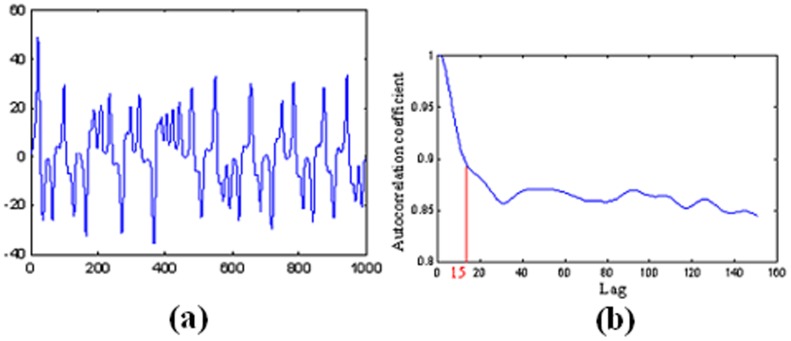
(a) Transient response of Y for σ = 10, τ = 50 and b = 8/3. (b) Part of the correlogram of the signal.

The Lorenz attractor is a set of three deterministic equations introduced by Lorenz [31], a meteorologist working on weather models, when he was studying the nonrepeatability of the weather patterns. The equations approximate the two-dimensional flow of a fluid heated along the bottom. The Lorenz attractor can be obtained by simultaneously solving the following equations:
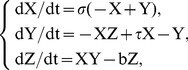
(13)which contain three model parameters. The Prandtl number σ, the relative Rayleigh number τ proportional to the applied difference in temperature and b the geometrical measure. Lorenz selected the values of 10 and 8/3 for σ and b respectively, to achieve a strong dissipate system, while emphasising that the use of slightly supercritical Rayleigh numbers may give realistic results [31]. Figure [5] (a) shows the transient response of Y over a finite number of observations for σ = 10, τ = 50 and b = 8/3, while Figure [5] (b) shows part of the correlogram of the signal. As it can be noticed, the rate of decrease of the autocorrelation coefficients starts to change at approximately lag 15 and the signal exhibits periodical behaviour.

The AE index is the auroral electrojet index determined from various stations located in the latitude region [32]. At these stations, the north-south magnetic perturbations are determined as a function of time and the superposition of the measured data determines two components, the maximum negative and the maximum positive excursion in the north-south magnetic perturbations. The difference between the two components is called the AE index [33].

**Figure 6:**
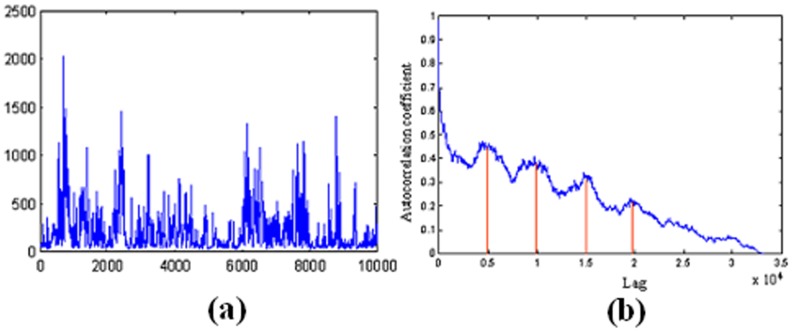
(a) The mean value of the AE index time series. (b) The correlogram of the mean value of the AE index signal.

The correlogram of the mean value of the AE index time series (refer to Figure [6] (b)) indicates that the autocorrelation coefficient drops to zero for large values of the lag. As a result, we can conclude that the time-series is a nonstationary signal. Furthermore, the signal shows periodicities for every 5000 lags.

There are various solar indices that can be used to express the activity of the sun. However, the International Sunspot Number (ISN) is considered one of the key indicators since the data is exceptionally lengthy and collected over a large number of years. The prediction of sunspot activity data is important for the space activity as well as the communication and the disaster prevention [41].

**Figure 7:**
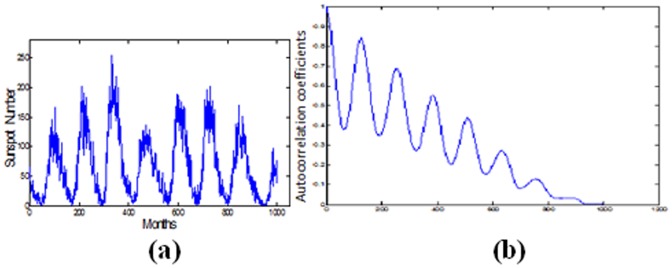
(a) The sunspot number time series from the year 1930 to 2013. (b) The correlogram of the signal.

Figure [7] (a) shows part of the sunspot time series while Figure [7] (b) shows the correlogram of the signal which clearly indicates that the signal is periodic and similar to the other physical signals, the correlogram goes to zero for a large value of the lag time.

**Figure 8:**
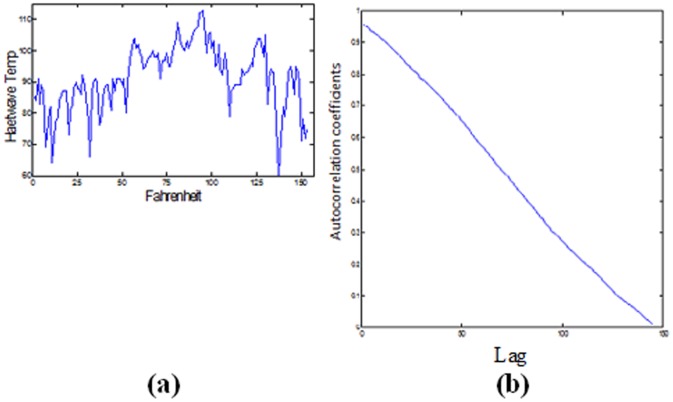
(a) The heatwave temperature time series. (b) The correlogram of the signal.

The Oklahoma City US daily heat wave temperatures for up to five months from May to September 2012 were used for the prediction task. The prediction is based on its pattern which is heat wave temperatures in Fahrenheit. The data was derived from the National Oceanic and Atmosphere Administration (NOAA, 2012). Figure [8] (a) shows the heat wave signal while Figure [8] (b) shows the correlogram of the signal which has no periodic as expected and goes to zero for a large value of the lag time which indicates the nonstationary property of the signal.

#### 3.2 Experimental Designs

The performance of the dynamic ridge polynomial neural network was benchmarked with the performance of the multilayer perceptrons (MLP), the functional link (FLNN), the pi-sigma (PSNN) and the ridge polynomial neural networks (RPNN). The prediction performance of the networks was evaluated using the normalised mean square of the error (NMSE) and the signal to noise ratio (SNR) matrices as shown in Table [1].

**Table 1:** Performance Metrics and their Calculations.

Metrics	NMSE	SNR
Calculations	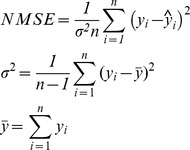	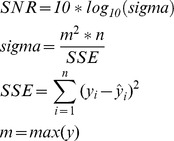

All the input and output variables were scaled in order to avoid computational problems and to meet algorithm requirements. A few reasons for using data scaling is to reduce the range difference in the data and to process outliers, which consist of sample values that occur outside the normal (expected) range. Furthermore, the data is scaled to accommodate the limits of the network's transfer function. Manipulation of the data using this process produces a new bounded dataset. The calculation for the standard minimum and maximum normalization method is as follows:
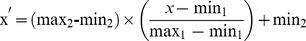
where 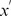 refers to the normalized value, x refers to the observation value (original value), min_1_ and max_1_ are the respective minimum and maximum values of all observations, and min_2_ and max_2_ refer to the desired minimum and maximum of the new scaled series.

The input-output variables were normalized between the interval [0.2, 0.8]. The choice of this interval is to avoid difficulty in getting network outputs too close to the two endpoints of Sigmoid transfer function.

The data sets used in this work were segregated in time order. In other words earlier period of data are used for training, and the data of the later period are used for testing. The main purpose of sorting them into this order is to discover the underlying structure or trend of the mechanism generating the data, that is to understand the relationship exist between the past, present and future data.

For the MLP, FLNN, and PSNN, each signal was divided into three data sets which are the training, validation and the out-of-sample which represent 25%, 25%, and 50% of the entire data, respectively. For the RPNN and DRPNN, the data were partitioned into two categories: the training and the out-of-sample data, with a distribution of 75% and 25%, respectively.

**Figure 9:**
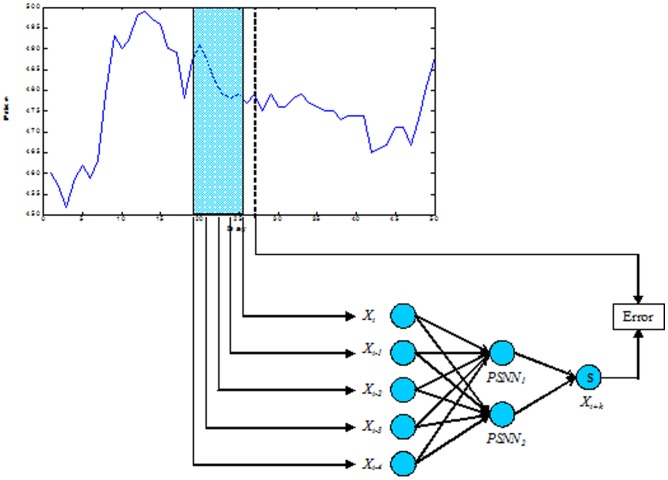
Neural network learns the non-stationary signal.

Figure [9] illustrates how the neural network is used to learn the non-stationary time series in which the previous values are used as input and the aim of the neural network is to predict the future values.

## Results

In this section, the simulation results for the prediction of the Lorenz attractor, the mean value of the AE index, sunspot number, and heat wave temperature using the dynamic ridge polynomial neural network will be presented.

The DRPNN was benchmarked with the MLP, FLNN and PSNN, which were trained with the incremental backpropagation learning algorithm [14]. Early stopping with maximum number of 3000 epochs was utilised. Each signal was divided into three data sets: the training, the validation and the out-of-sample data. The simulation results of the DRPNN were also benchmarked with the simulation results of the RPNN and the Linear Predictor Coefficient (LPC) model. For the training of the RPNN and DRPNN we partitioned the signals into two categories: the training and the out-of-sample data, as we did not employ early stopping.

For the DRPNN, we trained the network with a constructive learning algorithm as demonstrated previously. DPRNN provided the natural mechanism for incremental network growth. We started the network with order one, which had one block of Pi-Sigma Neural Network of order one. The training was carried out until the monitored error falls below the predefined threshold, which in this case *r*. At this time, a second order PSNN was added and the threshold, *r*, together with the learning rate, *n*, was decreased by a factor *dec_r* and *dec_n* respectively. The modified and updated network continues the learning and again, if the error fell below the threshold *r*, a higher order of PSNN block is added. This process was repeated until the maximum number of epochs was reached. Note that only the weights of the latest added Pi-Sigma unit were adjusted during the training and the rest were kept frozen.

For all neural networks, an average performance of 5 trials was used. The learning rate was selected between 0.1 and 0.5 and the momentum term was experimentally selected between 0.4 and 0.9. Two sets of random weight initializations were employed (in the range of [−0.5, 0.5] and [−1, 1]). Our primary interest is to assess the predictive ability of the DRPNN models against other neural networks and linear models, therefore, during generalization, we focus more on how the networks generate the prediction, and the neural network structure which endows the highest SNR on unseen data is considered the best model.

**Figure 10:**
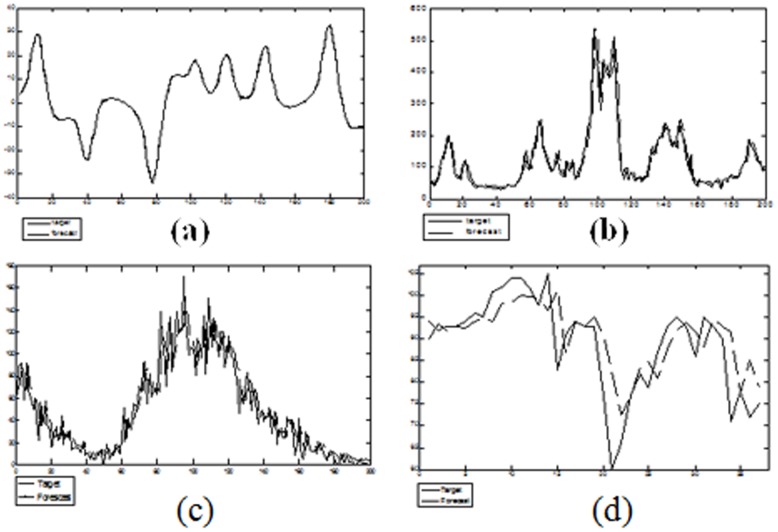
Part of the prediction for the (a) Lorenz attractor time series (b) the mean value of the AE index time series (c) sunspot number time series (d) heat wave temperature time series.

Table [2] shows the average performance over 5 simulations for the various neural network architectures and the linear predictor. Figure [10] shows part of the prediction of the Lorenz attractor, the mean value of the AE index, sunspot number, and heat wave temperature using the DRPNN.

## Discussion

As it can be witnessed from Table 2, all neural network architectures produced good simulation results for the prediction of the Lorenz attractor. There is a difference of 1.7 dB between the lowest average simulation produced by the PSNN and the best average simulation produced by the DRPNN. The average simulation results indicated that there is slight difference in performance between the third order FLNN and the third order DRPNN, however our simulation showed that the maximum number of trained epochs was necessary for the FLNN to achieve a good simulation results.

**Table 2:** The average simulation results over 5 trials using the benchmarked Neural Networks Structures and the simulation results using Linear Predictor Coefficient (LPC) model.

Lorenz attractor	MLP (Hidden nodes 3)	FLNN (Order 3)	PSNN (Order 2)	RPNN (Order 5)	DRPNN (Order 3)	LPC
NMSE	0.001924	0.001605	0.002356	0.001998	0.001606	0.0065
SNR (dB)	44.95	45.73	44.06	44.87	45.77	33.4022
Epochs	2860	3000	3000	1681	2496	N/A

The Mean Value of the AE index	MLP (Hidden nodes)	FLNN (Order 2)	PSNN (Order 2)	RPNN (Order 4)	DRPNN (Order)	LPC
NMSE	0.175805	0.133661	0.115797	0.068515	0.067267	0.0615
SNR (dB)	28.3	29.45	30.08	32.36	32.44	31.3648
Epochs	104	146	34	531	89	N/A

Sunspot Number	MLP (Hidden nodes 3)	FLNN (Order 3)	PSNN (Order 2)	RPNN (Order 4)	DRPNN (Order 3)	LPC
NMSE	0.1319	0.1366	0.132	0.1317	0.1301	0.1241
SNR (dB)	25.16	25.01	25.16	25.17	25.22	22.6233
Epochs	2134	1291	2338	2999	1443	N/A

Heatwave Temperature	MLP (Hidden nodes 3)	FLNN (Order 2)	PSNN (Order 2)	RPNN (Order 3)	DRPNN (Order 2)	LPC
NMSE	0.4938	0.4903	0.4888	0.4677	0.4713	0.8184
SNR (dB)	18.12	18.15	18.17	18.36	18.32	21.6947
Epochs	3000	2479	873	2357	243	N/A

Results from Table 2 also showed that the NMSE produced by DRPNNs on average is below 0.001 for the prediction of the Lorenz attractor. Despite the fact that the NMSE for DRPNNs when used to predict the Lorenz attractor signal is slightly higher than that of other FLNNs models, the results do not reflect the significant predictive value offered by DRPNNs. This is because we are more concerned with the out-of-sample prediction value of the network using the SNR rather than NMSE. The simulation results indicated that the linear predictor demonstrated the lowest value for the prediction of the Lorenz attractor signal using the NMSE and the SNR quality measures.

It should be pointed out that in this study the parameters of the dynamic ridge polynomial neural network architectures such as the number of inputs parameters, the momentum values, etc. where selected after a few trial and error tests on a limited number of parameter values. Since the results of the model with non optimal parameter values selection where significantly good indicating that the optimized neural network parameters will definitely lend the trained models as equally good as or even better performance than those of limitedly trained neural networks shown in our simulation results.

The simulation results for the prediction of the mean value of the AE index showed that the DPRNN demonstrated the best results using the SNR, while the MLP network illustrated the worst SNR values with 28.3 dB which is lower than the LPC predictor that demonstrated a SNR value of 31.3648.

The prediction of the sunspot signal illustrated that all neural network architectures achieved similar value for the SNR with approximately 25 dB. The linear predictor demonstrated again the lowest value of 22.6233 dB.

As it can be shown from Table 2, the prediction of heat wave signals indicated that the LPC predictor showed the best SNR, while all the neural network architectures failed to achieve a SNR above 20 dB. However, the NMSE for all the neural network architectures indicted better values than the NMSE achieved by the linear predictor.

**Figure 11:**
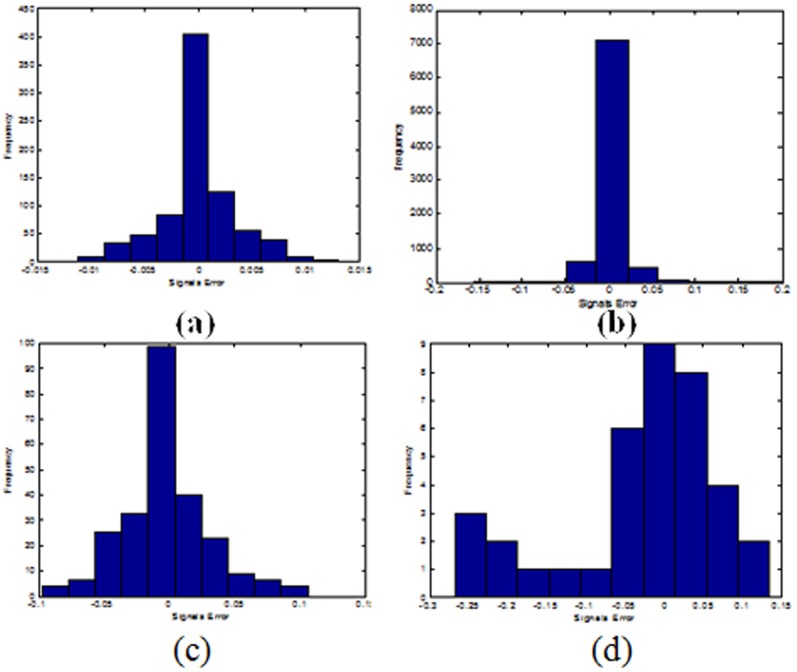
The histogram of the prediction error for (a) the Lorenz attractor time series; (b) the mean value of the AE index time series (c) sunspot number time series (d) heat wave temperature time series using the DRPNN.

As it can be noticed from Figure [11], the histograms of the nonlinear prediction errors for the Lorenz attractor, the mean value of the AE index and the sunspot signals using the DRPNN may be considered to show Gaussian distributions. This is an indication that the DRPNN managed to extract the information from the signal and hence the good simulation results. Figure [11] (d) shows the histogram of the error values for the prediction of the heat wave signal which indicted a random distribution and hence the network could not provide a good simulation value in terms of the SNR.

The use of Dynamic Ridge Polynomial Neural Networks in physical time series showed that the network provides a promising tool to forecasting. The network offers the following advantages:

- It provides better prediction in terms of the SNR in comparison to other neural network architectures. The prediction attained by the DRPNNs for the Lorenz attractor is slightly higher than that of FLNN but significantly better than the prediction generated by the PSNN, which is about 0.04 dB to 1.7 dB higher. For the prediction of the mean value of the AE index time series showed significantly improved results over the MLP and slightly better results than the RPNN, which is about 0.08 dB to 4.14 dB higher.
- In view of the fact that the behaviour of the physical signal is related to some past inputs on which the present inputs depends, it therefore requires explicit treatment of dynamics. The merit of DRPNN, as compared to the RPNN is its increased inherited nonlinearity which results from the use of recurrent neural networks architecture, giving it an advantage when dealing with time series forecasting.
- The Dynamic Ridge Polynomial Neural network demonstrated faster training when used to learn the Lorenz attractor signal in comparison to the MLP, FLNN and PSNN networks. For the prediction of the mean value of the AE index, the proposed network showed significantly faster training in comparison MLP, FLNN and RPNN.

**Figure 12:**
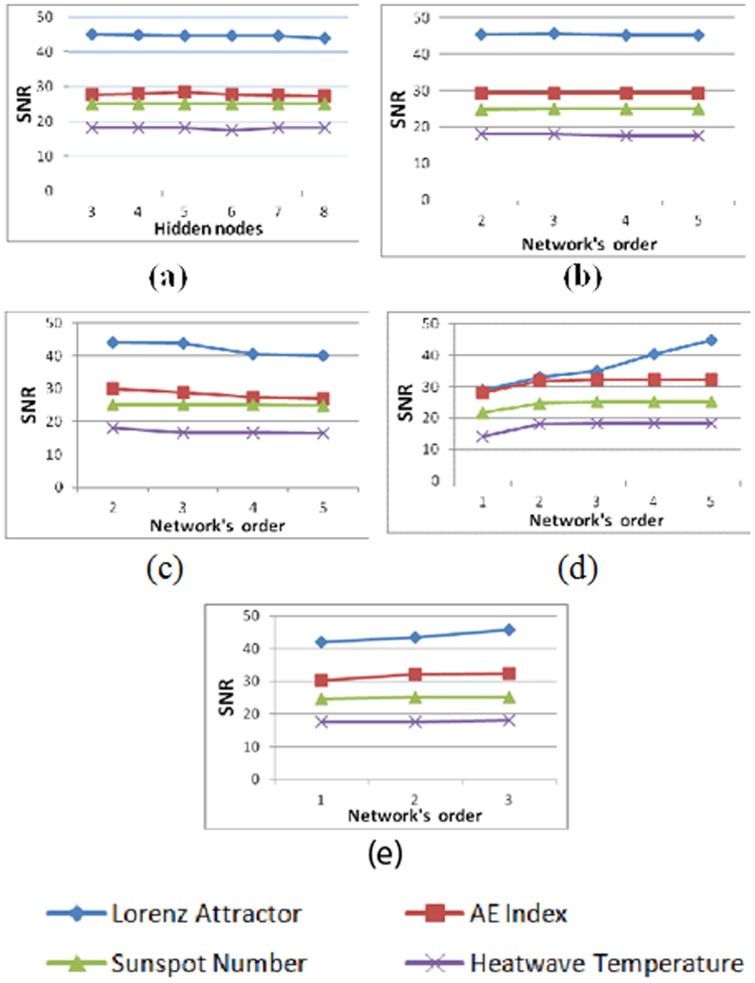
Performance of all networks with increasing order/number of hidden nodes (a) MLP, (b) FLNN, (c) PSNN, (d) RPNN, (e) DRPNN.

Figure [12] illustrates the signal to noise ratio from the best result tested on out-of-sample data when used to predict the Lorenz attractor and the mean value of the AE index. The performance of the networks was evaluated with the number of higher order terms increased from 1 to 5 for HONNs, and number of hidden nodes increased from 3 to 8 for MLP network. The plots in Figure [12] (a) and Figure [10] (b) indicate that the MLPs and the FLNNs, respectively, showed no increase in the value of the SNR for the two signals. However, for the prediction of Lorenz attractor and the mean value of the AE index using PSNN, the SNR started to decrease for a 3^th^ order PSNN network. This is probably due to the utilization of large number of free parameters for the network of order three and this has led to unpromising generalization for the input-output mapping.

On the other hand, the plots for RPNN and DRPNN in Figures [12] (d) and (e), respectively, shows that the networks have learned the data steadily with the SNR continues to increase along with the network growth.

**Figure 13:**
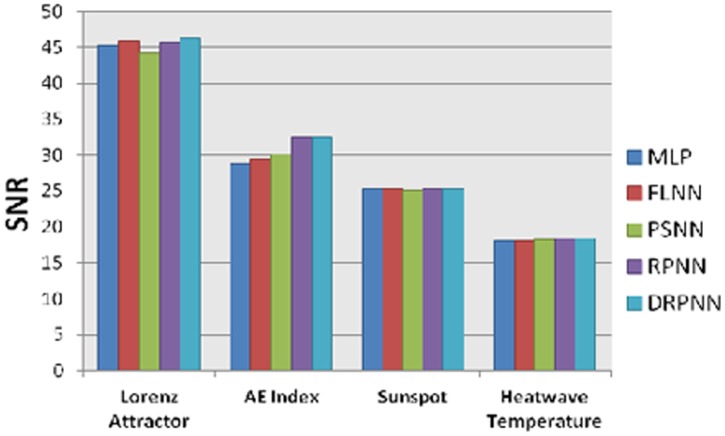
Best simulation result using the SNR.

Figure [13] shows the best simulation results for all neural networks for the prediction of the Lorenz attractor, the mean value of the AE index, sunspot number, and heat wave temperature which indicates that the DRPNN showed better simulation results using the SNR than the benchmarked networks for the prediction of the Lorenz attractor and similar results to the performance of the RPNN for the prediction of the mean value of the AE index.

In our simulation, we investigated a range of values for the parameters that influence the network performance with stable results. The results show the same performance figures sustained across different training and testing sets. The summary is as follows:

1. *The stability of the network with various network architecture*: In this simulation, we tested the network architecture by varying the number of pi-sigma units from 2 to 5. The results show that the signal to noise ratio stayed stable as illustrated in Figure [12].
2. *The stability of the network with the number of iterations:* In this experiment, the results remain stable for the training time between 100 and 3000 epochs. No indication of over training was noticed and the performance was improved with larger number of iterations. This behaviour is sustained for the all the time series used in the simulation.

## Conclusions

This paper investigated the predictive capability of the Dynamic Ridge Polynomial Neural Network, for the prediction of physical time series signals. The results were benchmarked with the Multilayer Perceptron and higher order neural networks, as well as linear predictor. Experimental results showed that DRPNNs produced improved performance in terms of the SNR. In addition to generating good performance, which is a desirable property in nonlinear time series prediction, DRPNNs also used smaller number of epochs during the training in comparison to the MLPs. This is obviously due to the presence of only a single layer of adaptive weights. The enhanced performance in the prediction of the physical time series using DRPNNs is due to the networks robustness caused by the reduced number of free parameters compared to the MLPs.

## Supporting Information

File S1 **lorenz_original.** The Lorenz attractor is a set of three deterministic equations that is used to create a simulated signal. The equations approximate the two-dimensional flow of a fluid heated along the bottom. The Lorenz attractor can be obtained by simultaneously solving the following equations.
(TXT)

File S2 **mae78_original.** The AE index is the auroral electrojet index determined from various stations located in the latitude region.
(TXT)

File S3 **sunspot.** The sunspot numbers data is a recording of observed sunspot activity over a period of time by the World Data Center for the production, preservation and dissemination of the international sunspot number.
(TXT)

File S4 **heatwave.** The heatwave data is a record of global temperatures over a period of a year by the US national oceanic and atmospheric administration.
(TXT)
